# Editorial: Affective self-regulation and mental disorders: A transdiagnostic disposition in psychopathology

**DOI:** 10.3389/fpsyt.2022.1053988

**Published:** 2022-12-01

**Authors:** Ilana S. Hairston

**Affiliations:** ^1^Psychology Department, Tel-Hai Academic College, Qiryat Shemona, Israel; ^2^The Institute of Information Processing and Decision Making (IIPDM), University of Haifa, Haifa, Israel

**Keywords:** Research Domain Criteria (RDoC), HiTOP classification, transdiagnostic analysis, emotion regulation, Affect

While a classification system of psychiatric disorders remains a necessary aspect of clinical psychiatric practice, comorbidity and shared illness trajectories are an obstacle to identifying etiologies of disorders, e.g., (1–3). This has led practitioners and researchers to propose a transdiagnostic view of psychiatric illness and therapy (3–7). As noted by Mansell et al. (5) and Nolen-Hoeksema and Watkins (7), the notion that mental disorders share cognitive, behavioral and emotional features and patterns of comorbidity is not new. In fact, in the early days of psychology and psychiatry, the dominant view was that shared processes, such as neuroses and defense mechanisms or conditioned responses, were common origins of psychopathologies. It was only in the latter half of the twentieth century that classification systems were developed that allowed practitioners to assign a patient with a particular pattern of signs and symptoms a specific diagnosis and treatment regimen.

However, it remained evident that disorders were heterogeneous, that different disorders shared dysfunctional processes, and that these processes had wide-ranging continua that could be observed in non-clinical samples as well (8). Moreover, in the absence of solid etiology, the diagnostic categories were largely descriptive in nature (9). This lack of clarity has led to an effort in recent years to formulate a transdiagnostic approach to improve assessment and treatment practices (10–14). A transdiagnostic approach tries to identify dysfunctional processes and characterize their expression in relation to other phenotypic phenomena, i.e., symptoms and signs, that play a role in the onset and/or maintenance of disorders. Thus, the focus has shifted from understanding the etiology and trajectory of disorder categories (e.g., depression, eating disorders) to focusing on environmental, social, cognitive, behavioral, physiological, emotional, and molecular processes and their co-occurrence. New dimensional systems, such as the Research Domain Criteria (RDoC) e.g., (11) and Hierarchical Taxonomy of Psychopathology (HiTOP) (13, 15) have been proposed that focus on continuously distributed traits theorized to form the framework for psychopathology.

In this context, the purpose of this Research Topic was to focus on manifestations of affective dysregulation that are common to a broad range of mental disorders. It has been proposed that the dys/regulation of affect is a mental domain that lies at the intersection of several levels of analysis, from the molecular to the psychosocial aspects of psychopathology (16, 17). The goal was to underscore fundamental psychological and/or behavioral tendencies that underlie disorders and may act as clear treatment targets independent of the specific diagnosis.

Golan, in this issue, reports on a prospective study of a treatment protocol for eating disorders, Eating and Control Styles Axis (ECOSA), a tool developed to improve awareness, acceptance, and evaluation of one's own adaptive interoceptive emotional experience over exteroceptive feedback to achieve emotional validation, emotional self-efficacy, and self-agency. A large body of evidence demonstrates that difficulties in differentiating, describing, and regulating emotions play an essential role in eating disorders (18, 19), and that individuals with eating disorders report a greater reliance on maladaptive emotion regulation strategies compared with healthy controls (20, 21). Specifically, the ability to down-regulate negative mood states and overvalue immediate gratification are key features across different eating disorders. People with eating disorders struggle with emotion-induced impulsivity (22) associated with lower resilience in the face of negative emotions and a tendency to disproportionately value immediate rewards (namely eating) (23). ECOSA focuses on the association between an individual's over and/or under-control coping styles and the influence on decisions taken while experiencing uncomfortable and intolerable feelings. In the study, 94 women with eating disorders were randomized into ECOSA protocol or mentalisation-based psychotherapy. While both groups demonstrated improvement, the ECOSA group demonstrated more significant improvement in reflective functioning than the control treatment group.

Within the context of impulse control, Rodríguez-Nieto et al. assessed the relationship between self-control, sexual compulsivity, frequency of sexual behaviors, and levels of testosterone in a non-clinical sample of young men. Whereas, sexual excitation and inhibition have been extensively studied in the context of compulsive sexual behavior, this study's novel approach is assessing the potential contribution of self-control and testosterone levels. They demonstrated that low self-control was negatively- and testosterone levels were positively associated with sexual compulsivity proneness. Interestingly, other than a weak negative correlation between testosterone levels and frequency of intercourse, sexual behaviors were not correlated to any of the measures, potentially indicating that behavioral expression of sexual compulsion is distinct from its cognitive aspects.

Impulse control is a central construct associated with the externalizing super-spectrum in the HiTOP system, which ranges from normative levels of impulses to extensive polysubstance involvement and personality psychopathology. The validity of this super-spectrum is supported by a large body of literature on externalizing behaviors such as disinhibition and antagonism (24). Indeed, research suggests that there is shared variance among the array of dysregulated behaviors, including sexual compulsive behaviors (25). Thus, this study expands our understanding of impulse control and the role of hormone levels in tendencies for sexual compulsion, consistent with the perspective of RDoC that mental disorders are on continua, and can be described on multiple levels, including biological (12).

Dollberg and Hanetz-Gamliel describe a cross-sectional study of Israeli mothers of children (ages 3–12) assessed during the month-long lockdown of the COVID-19 pandemic. They show that mothers' adverse childhood experiences (ACEs) predicted higher depressive and anxiety symptoms and children's internalizing and externalizing problems. Importantly, mothers' symptoms of depression and anxiety mediated the links between their ACEs and their children's internalizing behaviors. The spectrum of traumatic stress effects, from recalled trauma to posttraumatic stress disorder (PTSD), has received substantial attention within the RDoC framework. It has been hypothesized that this spectrum forms a dimensional construct that coalesces several behavioral tendencies, cognitive, personality, neurobiological, and environmental factors that encompass both negative and positive adaptations to stress, and whose effects are conveyed *via* developmental trajectories (26). Dollberg and Hanetz-Gamliel findings further highlight the transgenerational effects of traumatic experience, underscoring the importance of the environment dimension in the RDoC.

Hen et al. also focus on the COVID-19 pandemic as a stressor. The authors explore the impact of the COVID-19 pandemic on the mental health of children and adolescents (ages 8–17), comparing assessments of behavioral problems, anxiety, depressed moods, and difficulty in emotional regulation among children referred to an ambulatory clinic prior to the pandemic (*n* = 298) to children who were referred during the pandemic (*n* = 132). The study finds that during the pandemic, children reported more significant anxiety, more emotional and behavioral problems, and greater difficulty with emotion regulation. Moreover, while for girls these associations were associated with more depressive symptoms, for boys negative affective states were significantly associated only with anxiety. These findings resemble similar observations in non-clinical samples (27, 28), thus supporting the notion that the underlying dimensions of psychopathology lie in continua, which are similar across clinical and non-clinical samples.

Finally, Hairston et al. tested the association between positive and negative ruminations and subjective sleep, and the moderating role of reappraisal and suppression on this association, in a non-clinical sample of adults. It has recently been shown that repetitive thoughts regarding positive events are a trait associated with psychopathology (29–31), similar to negative rumination (32). This led the authors to ask whether the repetitive nature of thoughts or their valence were associated with symptoms of insomnia. The former predicting similar negative effects of both positive and negative rumination on subjective sleep, the latter predicting opposite effects, namely negative rumination interfering with sleep while positive rumination having null or a positive effect. The authors found that indeed positive rumination *per se* had a slight positive effect on sleep; however this effect was moderated by emotion regulation strategies, with reappraisal reversing the effects such that positive rumination interfered with sleep among respondents with higher levels of reappraisal.

These findings touch on various well-documented transdiagnostic features. Sleep and sleep disturbances have long been understood to be a transdiagnostic feature, with insomnia symptoms being comorbid with up to 60% of psychiatric diagnoses (33). Similarly, rumination, specifically negative repetitive thinking, represents a maintaining factor of emotional disorders, accompanying a variety of psychopathologies (34, 35); and as noted above, recent evidence suggests that positive rumination may also be involved in psychopathology. Moreover, emotion regulation strategies have been differentiated between two main categories of ER strategies: adaptive and maladaptive. Whereas adaptive strategies, such as reappraisal, seem to reduce the risk for psychopathology and improve coping with adverse events, maladaptive strategies, such as negative rumination and suppression are risk factors for emotional distress and dysfunctional (36, 37). Thus, the findings of Hairston et al. add to the perspective that different assemblages of symptoms and traits form unique behavioral and cognitive constellations, which should inform both research and intervention development.

In sum, these diverse studies demonstrate the importance of understanding the network of environment, symptoms, physiology, and personality in creating the pattern of symptoms (or syndrome) and that both treatment and prognosis need to be seen from a transdiagnostic perspective.

## Author contributions

The author confirms being the sole contributor of this work and has approved it for publication.

## Conflict of interest

The author declares that the research was conducted in the absence of any commercial or financial relationships that could be construed as a potential conflict of interest.

## Publisher's note

All claims expressed in this article are solely those of the authors and do not necessarily represent those of their affiliated organizations, or those of the publisher, the editors and the reviewers. Any product that may be evaluated in this article, or claim that may be made by its manufacturer, is not guaranteed or endorsed by the publisher.
